# Intraocular pressure modulation with thermal stimuli

**DOI:** 10.5935/0004-2749.2023-0083

**Published:** 2024-07-09

**Authors:** Thiago Carvalho Barros de Oliveira, Juliana de Lucena Martins Ferreira, Hissa Tavares de Lima, Carlos Otávio de Arruda Bezerra Filho, Joao Crispim Ribeiro

**Affiliations:** 1 Instituto Cearense de Oftalmologia, Fortaleza, CE, Brazil; 2 Department of Ophthalmology, Centro Universitário Christus, Fortaleza, CE, Brazil; 3 Department of Ophthalmology, Universidade Estadual do Ceará, Fortaleza, CE, Brazil

**Keywords:** Intraocular pressure, Temperature, Masks, Glaucoma, Eye diseases

## Abstract

**Purpose:**

This study aimed to determine whether early-stage intraocular pressure can be modulated using a thermal face mask.

**Methods:**

In this prospective clinical study, healthy participants were randomized on a 1:1:1 allocation ratio to three mask groups: hypothermic (G1), normothermic (G2), and hyperthermic (G3). After randomization, 108 eyes from 108 participants were submitted to clinical evaluations, including measurement of initial intraocular pressure (T1). The thermal mask was then applied for 10 minutes, followed by a second evaluation of intraocular pressure (T2) and assessment of any side effects.

**Results:**

The hypothermic group (G1) showed a significant reduction in mean intraocular pressure between T1 (16.97 ± 2.59 mmHg) and T2 (14.97 ± 2.44 mmHg) (p<0.001). G2 showed no significant pressure difference between T1 (16.50 ± 2.55 mmHg) and T2 (17.00 ± 2.29 mmHg) (p=0.054). G3 showed a significant increase in pressure from T1 (16.53 ± 2.69 mmHg) to T2 (18.58 ± 2.95 mmHg) (p<0.001). At T1, there was no difference between the three study groups (p=0.823), but at T2, the mean values of G3 were significantly higher than those of G1 and G2 (p<0.00).

**Conclusion:**

Temperature was shown to significantly modify intraocular pressure. Thermal masks allow the application of temperature in a controlled, reproducible manner. Further studies are needed to assess the duration of these effects and whether they are reproducible in patients with pathologies that affect intraocular pressure.

## INTRODUCTION

Intraocular pressure (IOP) is determined by the balance between the production and drainage of aqueous humor (AH), which, in turn, is determined by several chemical and biological processes. Several studies have investigated whether IOP can be modified using temperature^(1,2)^. Hyperthermia induction in rats showed increases in IOP. The measurements found that a 1.6°C increase in rectal temperature was correlated with an increase in AH flux of 126%. A direct link between corneal surface temperature and AH flux was also observed^(1)^. Other studies have shown that reductions in external temperature cause equivalent reductions in IOP^(2,3)^.

Several mechanisms have been proposed to explain this effect. This includes a temperature-induced increase in AH production with no facilitation of AH drainage caused by vascular modification in the anterior segment^(3)^. It has also been speculated that temperature fluctuations may induce both oxidative stress and stimulation of the sympathetic nerve fibers, altering the regulation of aqueous humor flow and production and thereby influencing IOP^(2)^.

Thermal masks can be applied to the orbital surface to change the temperature of the eye. They can be used as a form of cold compress for allergies or inflammation reduction, or as warm compresses to relieve eyelid diseases such as blepharitis^(4,5)^. However, to date, there has been no research on the use of thermal eye masks for IOP modulation. This study aimed to determine whether thermal masks can be used for IOP modulation.

## METHODS

A randomized, triple-blind clinical trial was conducted with adult patients, with a 1:1:1 allocation ratio to hyperthermic, normothermic, and hypothermic mask groups. The evaluations were carried out at the Instituto Cearense de Oftalmologia (ICO) in Fortaleza, Ceará, Brazil. Signed informed consent forms were obtained from all participants at the time of study enrollment after the nature and any possible consequences of the research had been explained to them. This study was conducted in line with the tenets of the 2013 revision of the Declaration of Helsinki and approved by the Ethics Committee of the Centro Universitário Christus (Unichristus) (protocol no. 38671320.5.0000.5049).

### Study population

The participants were selected from healthy individuals aged 20-80 years with no previous ocular or systemic pathologies who were attending the ICO for general evaluations. After agreeing to participate and completing the informed consent form, each volunteer responded to a brief questionnaire to verify that they met the inclusion criteria and did not meet the exclusion criteria of the study. The questionnaire included items about ocular trauma, ophthalmologic surgeries, and previous ocular pathologies. Individuals who met the criteria underwent a complete eye examination, which involved autorefractometry, a visual acuity test, anterior biomicroscopy, tonometry, and a retinal examination. Those with any of the predetermined exclusion criteria were identified during these assessments.

Volunteers who did not meet the exclusion criteria were randomized to one of the three mask temperature groups and a face mask was applied at the predetermined temperature for the relevant group. In each participant, both eyes were evaluated, but only the right eye was used for statistical analyses.

The inclusion criteria were adults who did not present with pathologies that modify the flow of AH. The exclusion criteria were diagnoses of glaucoma or cataracts, visual acuity worse than 20/30, IOP >21 mmHg, a cup-to-disc ratio >0.5; or a narrow anterior chamber angle. These were identified during ophthalmologic evaluation. Volunteers who wore contact lenses on the day of the evaluation were also excluded due to the unknown possible effects of their use on initial measurements or study outcomes table 1 shows the study demographics.

**Table 1:** Participant demographics

	**Cold mask (N = 36)**	**NT mask (N = 36)**	**Warm mask (N = 36)**	**Total**
Sex				
Male	21	17	18	56
Female	15	19	18	52
Mean age	51.01	52.2	51.7	
Age variation	(21-74)	(25-74)	(22-78)	
Ethnicity				
Latin	30	29	28	87
Black	4	5	5	14
White	3	2	3	8
Initial IOP	16.97±2.59 mmHg	16.50±2.55 mmHg	16.53±2.69 mmHg	

IOP= intraocular pressure; NT= normothermic.

After a thorough examination, a thermal mask at the temperature predetermined for their assigned group was applied to each participant for 10 minutes. The mask temperatures were obtained in accordance with the manufacturer’s recommendations.

### IOP measurement

This study took place during the COVID-19 pandemic. Therefore, considerable research was performed to determine which tonometer should be used in this research. Multiple contemporary investigations have documented the existence of SARS-CoV-2 within the tear film of patients with the virus. There is also an 18.2% prevalence of SARS-CoV-2 on the ocular surface, substantiating the plausibility of ocular transmission as an important consideration^(6)^.

There is some evidence that air-puff tonometers (APTs) release aerosols from the tear film, and there was an initial concern about the proliferation of SARS-CoV-2 through the use of these devices, studies have demonstrated positive results from polymerase chain reactions of the tear film in only 7.5% of confirmed cases, and the recommendation to avoid the use of APTs was withdrawn^(7,8)^. In this study, volunteers with IOP >21 mmHg were excluded to ensure that individuals at risk for glaucoma were not included in our sample. Therefore, an APT was considered viable for stable, reproducible evaluations of all participants. These are considered more acceptable for use during the pandemic than the Goldman applanation tonometer.

The primary outcome measure was changes in IOP measured in millimeters of mercury on a non-contact tonometer between initial IOP measurement (T1) and immediately after 10 min wearing a thermal mask (T2 tonometry). The time interval between the removal of the mask and the subsequent IOP measurement was roughly 20 s.

The secondary outcomes included differences in lOP modification between the three mask groups and side effects. In each participant, three measurements were taken before applying the mask and three immediately after its use. The average of each set of three was used in the study analyses.

### Thermal modulation of masks

Six commercial thermal masks (Thermogel) were utilized in this study. The assistant responsible for the application and modulation of the masks did not have access to the initial IOP measurements or participate in patient evaluation after the masks were removed. The masks were stored at an ambient temperature (approximately 28ºC). Two hours before each patient evaluation, two masks were placed in a refrigerator for cooling. Another two masks were maintained at an ambient temperature until the study, and the remaining two were heated in a microwave for 1 min and 30 s, per the manufacturer’s instructions, as needed. The information provided by the manufacturer indicated that the hypothermic masks reach a temperature of 5^o^C after 2 h of cooling. After 1 min and 30 s in the microwave, the hyperthermic masks reach approximately 55^o^C. A single test was conducted in another facility to determine if the temperatures were suitable and consistent with the information provided. Our data were consistent with the reported values. lt was not feasible to conduct these tests daily due to the lack of appropriate equipment at the study site.

### Statistical analysis

We used the mean reduction in the difference between the lOP of eyes treated or not treated with a cold mask (right eye: 10.01 ± 1.76 vs. 13.3 ± 1.25 mmHg; left eye: 11.33 ± 2.11 vs. 14.33 ± 3.78 mmHg) from a previous study to estimate that we needed to evaluate a minimum of five patients per group for the right eye, and 22 patients per group for the left eye, adopting 90% power and a 95% confidence interval^(2)^. As the sample size estimation for the left eye included both samples, 36 patients per group were evaluated to account for uncertainties in these assumptions.

For the primary analysis, a multiple linear regression model was used, with lOP pre- and post-mask application as the dependent variable and mask temperature as a covariant.

Data were expressed as mean and standard deviation, submitted to a Kolmogorov-Smirnov normality test, and compared using the Wilcoxon test for intragroup analyses and the Kruskal-Wallis/Dunn test for between-group analyses.

All analyses were performed using 95% confidence intervals on GraphPad Prism 5.0 software (GraphPad Software Inc., CA, EUA). In the primary analysis, p<0.05 was considered statistically significant. Due to the potential for type l errors due to multiple comparisons, the secondary outcome results were considered exploratory.

## RESULTS

**Figure 1:**
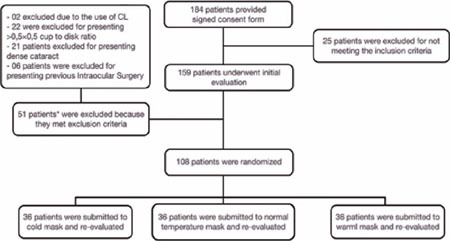
Randomization and patient selection flow diagram.

Between November 2020 and December 2021, 184 volunteers signed the consent form for study participation. Two were excluded due to the use of contact lenses before the evaluation. A further 49 participants were excluded due to previous ocular disease or surgery. The remaining 108 were randomly assigned to the three mask temperature groups. There were no significant differences in the main demographic characteristics of the three groups. A flow chart of the randomization and participant selection procedures is shown in figure 1.

### Main results

**Figure 2:**
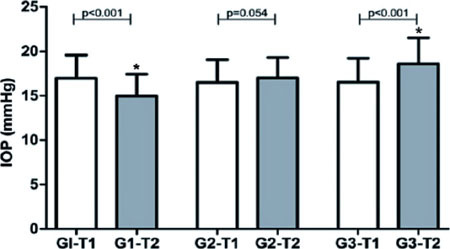
Comparison of intraocular pressure (IOP) between groups after the application of hypothermic, normothermic, and hyperthermic masks.

G1 showed a significant reduction in mean lOP from T1 (16.97 ± 2.59 mmHg) to T2 (14.97 ± 2.44 mmHg) (p<0.001). G2 showed no significant variation from T1 (16.50 ± 2.55 mmHg) to T2 (17.00 ± 2.29 mmHg) (p0.054). G3 showed a significant increase from T1 (16.53 ± 2.69 mmHg) to T2 (18.58 ± 2.95 mmHg) (p<0.001). At T1, there was no difference between the three study groups (p0.823) but, at T2, the mean value of G3 was significantly higher than those of G1 and G2 (p<0.001) (Figure 2).

**Figure 3:**
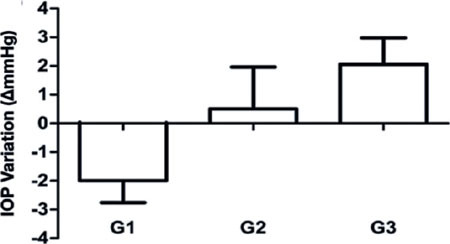
Comparison between intraocular pressure (IOP) variations before and after the application of hyperthermic, normothermic, and hypothermic masks.

In our comparison of the mean lOP variation between the three groups, G1 (−2.00 ± 0.76 mmHg) showed a significant reduction compared to G2 (+0.50 ± 1.46 mmHg), which, in turn, had significantly lower values than G3 (+ 2.06 ± 0.92 mmHg) (p<0.001) (Figure 3).

### Adverse effects

The side effects reported by the three groups were heterogeneous. Among the groups, the most prevalent complaint was facial flushing, which was present in 20 participants in total, two in G1 and 18 in the G3 group. Pruritus was the second most common effect and was reported by eight participants, three in G1 and five in G3. Mild facial pain was reported by four volunteers, two in G1 and two in G3.

## DISCUSSION

In this clinical trial comparing the IOP changes in three groups before and after the application of thermal masks of different temperatures, we found an average IOP reduction of approximately 2 mmHg after mask use in G1, the hypothermic mask group. This variation was statistically significant and corresponded to a 15%-20% reduction in IOP between baseline and after mask use. This is compatible with the findings of other authors^(2)^.

Several physiological factors seem to be involved in the IOP reduction mechanism. One of these is the regulation of AH secretion and drainage^(1,3)^. AH is synthesized in a three-step process by ciliary body cells. The initial step depends on blood flow and the pressure gradient between systemic blood pressure and the ciliary interstitium^(9)^. By modifying the temperature in the anterior segment of the eye, we caused vascular changes and changes in the metabolic processes of the ciliary body and cornea. With the hypothermic masks, this comprised vasoconstriction and a reduction in metabolic processing. With the hyperthermic masks, this comprised vasodilation and an increase in metabolic processing. This was apparent in G3, the hyperthermic group, in which there was a mean increase in IOP between T1 and T2 of 2 mmHg. A change in drainage may also be induced by changes in temperature.

At high temperatures, there is an increase in oxidative stress and the production of endothelins-1^(10,11)^. Endothelins reduce trabecular meshwork motility. This affects aqueous humor drainage and IOP regulation, primarily by inducing local vasoconstriction^(12,13)^. The sudden increase in AH production due to vasodilation and the metabolic increases associated with reduced drainage could be responsible for the increase in IOP induced by the hyperthermic masks^(13)^.

G2 showed no statistically significant variation between T1 (16.50 ± 2.55 mmHg) and T2 (17.00 ± 2.29 mmHg) (*p*=0.054). This normothermic group was used as the control in this study and the lack of change allowed us to rule out the possibility of IOP changes due to mechanical factors such as anterior segment compression or eyelid closure.

Both intervention groups, G1 and G3, experienced mild side effects during thermal mask use, primarily facial flushing and itching. However, these effects were sufficiently mild to not affect the possible use of the masks for IOP modulation. The effects of hyperthermia on the vessels of the face can lead to vasodilation and facial flushing, which begins seconds after mask contact and resolves within minutes of mask removal^(14,15,16)^.

Our study had several limitations. Initially, we were unable to gauge the temperature of each mask prior to its application, as the thermal scanner was unavailable for the entire duration of the experiment. Consequently, we opted to employ a consistent method of heating or freezing for all mask groups. As the principal aim of our study was to assess the ability of the masks to modulate intraocular pressure (IOP), we concentrated on the transient impact of the masks and did not evaluate the long-term sustainability of their effects.

The mean difference in IOP between G1 and G3 at T2 was 4 mmHg, demonstrating a correlation between temperature application and IOP modulation.

Glaucoma is one of the leading causes of blindness worldwide, and IOP is currently the only treatable risk factor^(17,18,19)^. This study found a correlation between temperature and IOP, and temperature modification can modulate this fluctuation, at least in the short term. The ability of hypothermic masks to reduce IOP offers a potential means of reducing IOP but further studies are necessary to determine the length of this effect.
